# XRCC1 polymorphisms and breast cancer risk from the New York Site of the Breast Cancer Family Registry: A family-based case-control study

**DOI:** 10.4103/1477-3163.62535

**Published:** 2010-04-16

**Authors:** Jennifer Zipprich, Mary Beth Terry, Paul Brandt-Rauf, Greg A. Freyer, Yuyan Liao, Meenakshi Agrawal, Irina Gurvich, Ruby Senie, Regina M. Santella

**Affiliations:** Department of Environmental Health Sciences, Mailman School of Public Health, Columbia University, New York, NY, USA; 1 Department of Epidemiology, Mailman School of Public Health, Columbia University, New York, NY, USA

**Keywords:** Breast cancer, DNA repair, XRCC1

## Abstract

**Background:**

XRCC1 is a scaffold protein involved in the early and late stages of Base Excision Repair (BER). Three DNA polymorphisms occur in XRCC1, resulting in non-synonymous amino acid changes, which could alter the binding or regulatory activities of XRCC1.

**Materials and Methods:**

We used a family-based case-control study design to evaluate the association between XRCC1 polymorphisms and breast cancer risk. Participants were breast cancer cases and their unaffected sisters enrolled in the New York Site of the Breast Cancer Family Registry. Conditional logistic regression was used to assess associations between genotype and breast cancer. XRCC1 mRNA levels and DNA nicking activity were measured in lymphoblastoid cell lines from unaffected sisters to determine whether the XRCC1 R399Q polymorphism has a functional effect on expression or protein activity.

**Results:**

XRCC1 194W was associated with a non-significant increase in breast cancer, while XRCC1 280H and XRCC1 399Q were associated with a non-significant decrease in breast cancer. We found a significant increase in XRCC1 expression in 399Q/Q lymphoblastoid cell lines from unaffected sisters (n=28, P=0.03). An increase in median nicking activity was not statistically significant.

**Conclusions:**

Our results suggest that XRCC1 399Q may alter mRNA expression and DNA repair phenotype, although the main effects of the genotype were not significantly associated with familial cancer risk. Additional research on the regulation of XRCC1 expression will contribute to an understanding of how this polymorphism may impact disease risk.

## INTRODUCTION

X-Ray Cross Complementing-1 (XRCC1) was first cloned in 1990 by Thompson *et al.*, and the gene product was found to complement a previously described CHO cell line, EM9, which exhibited hypersensitivity to alkylating agents, X-rays, ultraviolet irradiation and defects in single-strand break repair.[1,2] Since its discovery, XRCC1 has been found to play a significant role in the early and late stages of BER by mediating protein interactions and regulating the activities of many BER proteins including hOGG1, hNTH1, hNEIL2, and MPG, PARP-1, DNA Polβ, and DNA Ligase III.[3–9]

Several gene polymorphisms in XRCC1 result in non-synonymous amino acid substitutions. There are three well-known XRCC1 polymorphisms located in or near important protein domains. The XRCC1 C→T polymorphism at codon 194 occurs near the N-terminal domain and results in an arginine (R) to tryptophan (W) substitution, the G→A polymorphism at codon 280 is near the BRCT I domain and results in an arginine (R) to histidine (H) substitution, and the G→A polymorphism at codon 399 is in the highly conserved BRCT I domain (exon 10), resulting in the substitution of an arginine (R) with a glutamine (Q).

Cellular phenotyping studies suggest repair-related deficits in the form of sister chromatid exchange, sensitivity to ionizing radiation and DNA adduct removal associated with XRCC1 399Q.[10–15] CHO cells expressing XRCC1 280H accumulated DNA single-strand breaks compared to wild type after exposure to H_2_O_2_ or MMS.[16] The XRCC1 194R allele has been associated with an increase in DNA strand breaks after exposure of lymphoblastoid cells to bleomycin or benzo(a)pyrene diol epxoide.[17]

Numerous epidemiologic studies have been conducted to evaluate the association between XRCC1 and breast cancer.[18,19] The findings have been inconsistent; the rare allele of each polymorphism (XRCC1 194W allele, XRCC1 280H allele, XRCC1 399Q allele) has been associated with either an increase or decrease in breast cancer risk depending upon the population or subgroup analyzed.

Despite the number of genetic epidemiology studies that have evaluated breast cancer risk in relation to XRCC1 genotype, few have focused on women at high risk due to family history. Family history is associated with a twofold or greater increase in the risk of breast cancer in a first-degree relative.[20,21] This association may be increased or enhanced by factors such as young age at diagnosis, the presence of a BRCA mutation, and the number of first and second-degree relatives with breast cancer.[20,21] It has been previously shown that reduced DNA repair capacity is associated with an increase in breast cancer risk even in women at elevated risk due to family history.[22,23] Therefore, we aimed to determine whether XRCC1 polymorphisms are associated with breast cancer in high-risk women. We further explored potential functional effects of the XRCC1 R399Q polymorphism using lymphoblastoid cell lines from unaffected sisters.

## MATERIALS AND METHODS

The subjects for this study were selected from families participating in the Metropolitan New York Breast Cancer Family Registry, one site of the Breast Cancer Family Registry (BCFR). The description of the resources, the recruitment, data collection methods of the BCFR is detailed elsewhere.[24] Briefly, BCFR is a multi-center study designed to address questions related to the genetic epidemiology of breast cancer. A total of 1,336 families including 4,871 individuals were recruited from clinical settings in the New York metropolitan area. There are 348 families with at least two sisters discordant for breast cancer (n=842 individuals); 90% donated a blood sample (754 individuals, 313 sister sets). In the present study we have included 693 subjects belonging to 278 sister sets that were available for genotyping.

Subjects were administered a questionnaire that collected information on demographics, personal history of cancer, pertinent lifestyle and environmental factors (ionizing radiation exposure, smoking, and alcohol consumption), and reproductive factors known to be significant in cancer development (pregnancy history, breastfeeding, hormone use). The family history of all cancers (excluding non-melanoma skin cancers, and cervical carcinoma *in situ*) were self-reported as was treatment for breast and ovarian cancers. We did not explore potentially interesting gene-environment interactions due to our limited sample size.

Three SNPs in XRCC1 were genotyped: R399Q (rs25487), R280H (rs25489) and R194W (rs1799782). Genomic DNA was genotyped using the fluorescence polarization method with commercially available fluorescently-labeled dideoxynucleotides (Acycloprime Fluorescence Polarization SNP Kit, Perkin Elmer Life Sciences, Boston, MA) described by Chen.[25] The XRCC1 399 and 194 primers, probes, and cycling conditions were as described previously.[26]

The forward and reverse primers for XRCC1 280 polymorphism were designed according to the human XRCC1 gene sequence (GenBank accession no. L34079). The primer and probe are as follows: forward primer CCC CAG TGG TGC TAA CCT AAT, reverse primer GGT CCA GTC TGG CCG ATA CCT, and probe ACT GGG GCT GTG GT GGG GTA. Each amplification reaction contained 25 ng of genomic DNA, 1xPCR Reaction buffer with MgCl2 (Roche Applied Science), 2 pmoles each of the forward and reverse primers (Invitrogen), 2 mM dNTPs (Roche Applied Science), and 1U of Taq polymerase (Roche Applied Science). The cycling conditions for XRCC1 R280H are: denaturing at 94°C for 45s, annealing at 64.6°C for 45s, and extension at 72°C for 60s.

The genotyping call rates ranged from 93 to 99%, and the missing epidemiologic data ranged from 31 to 50 observations depending upon the variable. In sum, the number of subjects available for analysis was: XRCC1 R194W (n=613), R280H (n=628), and R399Q (n=626). Replicate samples for all genotypes were randomly placed in the plate. Kappa values were ≥ 0.99 and all genotypes were in Hardy-Weinberg equilibrium.

The lymphoblastoid cell lines (LCLs) used in this study were immortalized as described previously[22,27] LCLs were selected from unaffected siblings, and were matched by age and race in each of the XRCC1 399 genotype groups. LCLs were wildtype at the remaining XRCC1 polymorphic loci (194 and 280). Nuclear extracts were prepared from LCL using the NE-PER Nuclear and Cytoplasmic extraction reagent (Pierce Biotechnology, Milwaukee, WI). Nuclear extracts were buffer exchanged into 10mM Tris-HCl, 200mM KCl, 1mM EDTA, 20% glycerol using Zeba desalt spin columns (Pierce Biotechnology, Milwaukee, WI) and stored at -80°C. Protein concentration was measured using a BCA Kit (Sigma, St. Louis, MO).

A 26-bp polyacrylamide gel electrophoresis (PAGE) purified uracil containing oligonucleotide (5'-GAT CAG GTA U*CC ATG GCG CCT TGC A-3') was used as a model substrate for the *in vitro* BER assay (Integrated DNA Technologies). The oligonucleotide was end-labeled with 50μCi γ-^32^P ATP (Perkin Elmer, Specific Activity 3000Ci/mmole) and 10 units of T4 PNK (NEB) for 30 min at 37°C and then column-purified with a Sephadex G-25 column (GE Healthcare Life Sciences) to remove unincorporated γ-^32^P ATP. The labeled substrate was then annealed with its complementary oligonucleotide in 100 mM KCl, 10 mM Tris-HCl pH 7.8, and 1 mM EDTA.

Each base excision repair reaction contained 0.5 pmoles of labeled substrate, 100 ng of nuclear extract, 40 mM HEPES-KOH, pH 7.8, 75 mM KCl, 2 mM DTT, 1 mM EDTA, 100 ng/μl BSA. Reactions were incubated for 30 min at 37°C and stopped by adding 3 ul of formamide loading buffer and placing the reactions on dry ice. The samples were heated at 95°C for 5 min, and placed on ice. The repair reactions were resolved on a 15% acrylamide-8 M urea gels (BioRad). The gels were fixed in 50% methanol, 15% acetic acid for 30 min at room temperature, and dried for 1.5 h at 80°C.

The dried gels were placed in a GE Healthcare phosphor storage screen for 2 h and were scanned using a Molecular Dynamics STORM Phosphorimager. Band intensity was quantitated using ImageQuant software; the band intensity for each reaction was divided by the band intensity of the uracil DNA glycosylase-generated positive control. The results are expressed as proportion of substrate cut. The C.V. was 13% as calculated based upon 10 experiments with the nuclear extract from a control lymphoblastoid cell line.

RNA was extracted from lymphoblastoid cell lines using an RNeasy Mini Kit (Qiagen, Valencia, CA). RNA was quantitated by UV spectrophotometry and the quality was ascertained by gel electrophoresis. cDNA was synthesized using oligodT primers with the Superscript First Strand Synthesis kit (Invitrogen, Carlsbad, CA). RT-PCR reactions were run using an Applied Biosystems Instruments 7500 (Applied Biosystems, Foster City, CA). Power SYBR Green PCR Master Mix was used for the PCR amplification (Applied Biosystems). The primer sequences are as follows: for XRCC1, forward GAT TCT GGG GAC ACA GAG GA, REVERSE AGG GAA CTC CCC GTA AAG AA, cyclophilin, forward GGT GAC TTC ACA CGC CAT AAT, reverse AAA CGC TCC ATG GCT TCC ACA, and β-actin, forward CCT CGC CTT TGC CGA and reverse TGG TGC CTG GGG CG. The cycling conditions for all of the primer sets were: 94°C for 10 min, 94°C for 15 s and 60°C for 45 s for 40 cycles. A dissociation curve was produced after each run to verify the precision of the amplification.

Multivariable conditional logistic regression was used to assess the association between XRCC1 variants, and breast cancer risk. Records with missing covariate data were excluded from the analysis. Confounding was assessed by a 10% change in the β-estimate observed upon the addition of the potential confounder into the model. For XRCC1 R399Q both dominant and codominant models are shown. For XRCC1 R280H and R194W only dominant models are shown due to the low Minor Allele Frequency. mRNA levels and DNA repair activity in the lymphoblastoid cell lines were analyzed with Kruskal-Wallis rank test.

SAS version 9.12 was used for all analyses.

## RESULTS

Our data suggests that the XRCC1 194W allele is associated with an increase in breast cancer risk, while XRCC1 399Q and 280H alleles are associated with a decrease in risk in women with a family history of breast cancer. There were no confounders identified for XRCC1 399 in the dominant models. In the age-adjusted and unadjusted models, XRCC1 399Q was associated with a marginally significant decrease in breast cancer risk in the dominant model (XRCC1 399 R/Q + Q/Q vs. R/R, unadjusted OR: 0.64, 95% CI: 0.41-1.00; age-adjusted OR: 0.65, 95% CI: 0.42-1.02.XRCC1 399Q was also associated with a marginally significant decrease in risk in the co-dominant models (XRCC1 399 Q/Q vs. R/R, age-adjusted OR: 0.44, 95% CI: 0.20-0.96; multivariable-adjusted OR: 0.44, 95% CI: 0.18 - 1.11). None of the remaining XRCC1 genotypes were significantly associated with breast cancer after including confounders [Table 1].

**Table 1:** *XRCC1* and breast cancer from the New York Site of the Breast Cancer Family Registry

XRCC1 polymorphism	Genotype	Cases N (%)	Non-cases N (%)	OR (95% CI)*	Cases N (%)	Non-cases N (%)	OR (95% CI)**
	CC	227 (84)	282 (86)	Reference	212 (83)	263 (86)	Reference
R194W	CT, TT	44 (16)	45 (14)	1.26 (0.69-2.28)	42 (17)	42 (14)	1.17 (0.61-2.25)
	GG	250 (92)	303 (91)	Reference	249 (92)	300 (91)	Reference
R280H	GA, AA,	23 (8)	29 (9)	0.94 (0.43-2.03)	22 (8)	28 (9)	0.87 (0.38-2.00)
	GG	126 (46)	139 (43)	Reference	126 (46)	139 (43)	Reference
R399Q	GA, AA	145 (54)	184 (57)	0.65 (0.42-1.02)	145 (54)	184 (57)	0.64 (0.41-1.00)
	GG	126 (47)	139 (43)	Reference	119 (47)	132 (44)	Reference
R399Q	GA	115 (42)	141 (44)	0.68 (0.43-1.07)	107 (42)	131 (43)	0.65 (0.39-1.10)
	AA	30 (11)	43 (13)	0.44 (0.20-0.96)	28 (11)	38 (13)	0.44 (0.18-1.11)

* Adjusted for age

** XRCC1 R194W is adjusted for menopause and age; XRCC1 R280H is adjusted for age at menarche, BMI, and age; no confounders were identified for XRCC1 R399Q dominant model, co-dominant model with XRCC1 R399Q is adjusted for menopause and age

**Figure 1:**
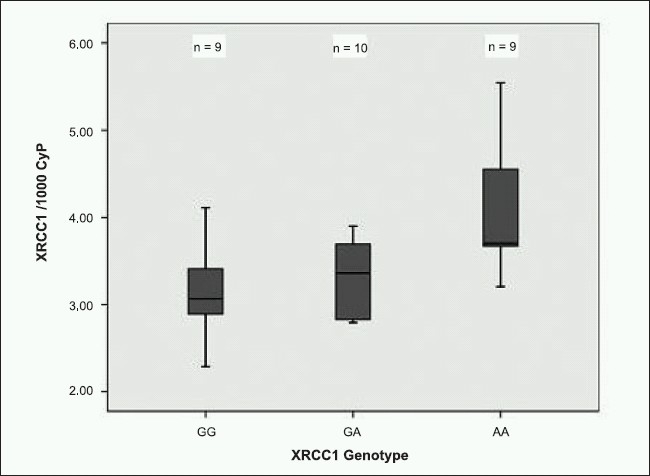
Boxplot of mRNA levels by XRCC1 genotype. Description: cell lines homozygous for the XRCC1 common variant (GG, R/R), heterozygous, or homozygous for the XRCC1 rare variant (AA, Q/Q) (Kruskal-Wallis Rank Test; Overall: *P*=0.03; pair-wise: GG vs. AA, *P*=0.02; GG vs. GA, *P* = 0.7; AA vs. GA, *P*=0.04).

Thirty LCLs, 10 expressing XRCC1 399R, 10 expressing XRCC1 399Q and 10 heterozygotes were selected to test for gene expression and DNA repair. XRCC1 mRNA levels by XRCC1 R399Q group are shown in Figure 1. For this experiment we excluded two cell lines, one for which we had no nuclear extract and another for which the nuclear extract was of poor quality. Both cyclophilin and β-actin were used as housekeeping genes to adjust XRCC1 mRNA levels. XRCC1 mRNA expression was significantly higher in XRCC1 399Q-expressing cell lines (cyclophilin-adjusted, Kruskal-Wallis, *P*=0.03; Actin-adjusted, Kruskal-Wallis, *P*=0.06, data not shown).

**Figure 2:**
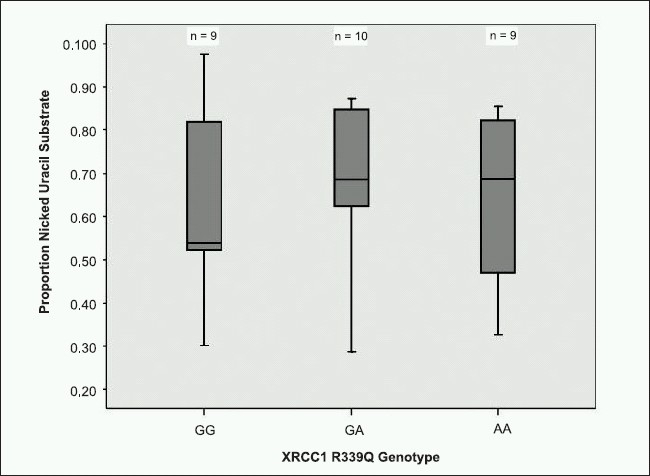
Boxplot of the proportion of nicked uracil substrate by XRCC1 genotype. Description: Nuclear extracts derived from cell lines homozygous for the XRCC1 common variant (GG, R/R), heterozygous, or homozygous for the XRCC1 rare variant (AA, Q/Q). Kruskal-Wallis Rank Test, *P*=0.96, Median One-way analysis, *P*=0.89.

To determine whether increased XRCC1 expression would translate into an increase in repair activity, we used a 28 - bp uracil-containing oligonucleotide as a model substrate to measure BER activity. Nicking activity in nuclear extracts from XRCC1 R399Q cell lines is shown in Figure 2. The median proportion of nicked substrate was lower in XRCC1 399R nuclear extracts (0.53) when compared with nuclear extracts prepared from XRCC1 399R/Q (0.67) and XRCC1 399Q/Q (0.69) cell lines. However, these differences were not statistically significant.

## DISCUSSION

The results from our epidemiologic analysis suggest that the rare allele of XRCC1 R194W increases breast cancer risk, while the rare alleles of XRCC1 R280H and XRCC1 R399Q decrease risk among women from high-risk families, though these findings were only marginally significant with XRCC1 399Q. A study from the Ontario site of the BCFR, which enrolled both familial and population-based breast cancer cases, observed an interaction between positive family history and the XRCC1 399Q allele.[28] Women in the XRCC1 399R group with a family history were found to have an increase in breast cancer risk, while women in the XRCC1 399Q group with a family history were found to have decreased risk when compared to women without a family history of breast cancer.[28] However, these findings were not statistically significant. Other studies have found no effect of family history, or have detected an increase in breast cancer risk associated with the XRCC1 399Q allele.[29–31]

To explore the possible functional effect of the XRCC1 R399Q polymorphism we measured XRCC1 gene expression and DNA nicking activity of lymphoblastoid cell lines prepared from unaffected sisters. The bioinformatics tool Regulatory Analysis of Variation in Enhancers (RAVEN) predicted that the XRCC1 R399Q polymorphism may overlap with a putative transcription factor binding site. According to RAVEN the XRCC1 sequence CCC**G**GAGGT containing the G allele (XRCC1 399R) was predicted to be a better binding site compared with the same sequence containing the A allele (XRCC1 399Q), suggesting that there may be expression differences between the two forms of XRCC1. We found that cell lines homozygous for the A allele (XRCC1 399Q) expressed XRCC1 at significantly higher levels than lines homozygous for the G allele (XRCC1 399R). Our findings are consistent with those reported by Milani *et al.*, where a 1.8-fold increase in the expression of XRCC1 mRNA from the A allele of XRCC1 compared with the G allele in heterozygous doxyrubicin-sensitive myeloma cells was observed.[32]

DNA nicking activity of a uracil substrate measures the initial recognition and endonuclease cleavage of the damaged nucleotide. Thus, less nicking could indicate a decrease in repair activity. Although we detected an increase in median nicking activity, this increase was not statistically significant. Perhaps more cell lines would have enhanced detection of subtle differences in nicking activity or the effect may have been stronger with a different DNA lesion such as an 8-oxodG.

## CONCLUSIONS

In sum, we report a significant increase in XRCC1 mRNA in XRCC1 399Q-expressing LCLs. We did not detect a significant increase in repair activity and the main effects of these genotypes were not significantly associated with breast cancer risk within families. These results generate questions regarding the regulation of XRCC1 expression and whether changes in transcriptional regulation underlie phenotypes previously reported to be associated with XRCC1 399Q.

## AUTHOR'S PROFILE

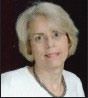



**Dr. Regina Santella's** research involves the development of laboratory methods for the detection of human exposure to environmental and occupational carcinogens and their use in molecular epidemiology studies to identify causative factors, susceptible populations, and preventive interventions. Her work has allowed the determination of exposure to carcinogens by the measurement of their binding to DNA with highly specific and sensitive immunoassays using monoclonal and polyclonal antibodies that her laboratory has developed. These studies have demonstrated higher levels of DNA damage in those with environmental or occupational exposures and in subjects with breast, lung, and liver cancer compared to controls. In addition, the interaction between environmental exposures and genetic susceptibility on cancer risk is being investigated using high throughput genotyping to determine polymorphisms in carcinogen metabolism, oxidative stress, and DNA repair genes.
